# Cardio-Oncology in the COVID Era (Co & Co): The Never Ending Story

**DOI:** 10.3389/fcvm.2022.821193

**Published:** 2022-01-28

**Authors:** Irma Bisceglia, Maria Laura Canale, Giuseppina Gallucci, Fabio Maria Turazza, Chiara Lestuzzi, Iris Parrini, Giulia Russo, Nicola Maurea, Vincenzo Quagliariello, Stefano Oliva, Stefania Angela Di Fusco, Fabiana Lucà, Luigi Tarantini, Paolo Trambaiolo, Antonella Moreo, Giovanna Geraci, Domenico Gabrielli, Michele Massimo Gulizia, Fabrizio Oliva, Furio Colivicchi

**Affiliations:** ^1^Integrated Cardiology Services, Cardio-Thoracic-Vascular Department, Azienda Ospedaliera San Camillo Forlanini, Rome, Italy; ^2^Cardiology Department, Nuovo Ospedale Versilia Lido di Camaiore Lucca, Camaiore, Italy; ^3^Cardio-Oncology Unit, Istituto di Ricovero e Cura a Carattere Scientifico (IRCCS), Referral Cancer Center of Basilicata, Rionero in Vulture, Italy; ^4^Cardiology Department, Fondazione IRCCS Instituto Nazionale dei Tumori, Milan, Italy; ^5^Azienda Sanitaria Friuli Occidentale (ASFO) Cardiac and Cardio-Oncologic Rehabilitation Service at Centro di Riferimento Oncologico (CRO), National Cancer Institute, Aviano, Italy; ^6^Cardiology Department, Ospedale Mauriziano Umberto I, Turin, Italy; ^7^Cardiovascular and Sports Medicine Department, Azienda Sanitaria Universitaria Giuliano Isontina (ASUGI) Trieste, Trieste, Italy; ^8^Cardiology Department, G. Pascale National Cancer Institute Foundation (IRCCS), Naples, Italy; ^9^Cardio-Oncology Department, Istituto Tumori Giovanni Paolo II, Bari, Italy; ^10^Clinical and Rehabilitation Cardiology Department, Ospedale San Filippo Neri, Rome, Italy; ^11^Cardiology Department, Grande Ospedale Metropolitano Bianchi Melacrino Morelli, Reggio Calabria, Italy; ^12^Divisione di Cardiologia, Arcispedale S. Maria Nuova, Azienda Unità Sanitaria Locale-IRCCS di Reggio-Emilia, Reggio Emilia, Italy; ^13^Cardiology Department, Sandro Pertini Hospital, Rome, Italy; ^14^A. De Gasperis Cardio Center, Aziende Socio Sanitarie Territoriali (ASST) Grande Ospedale Metropolitano Niguarda, Milan, Italy; ^15^Cardiology Unit, Azienda Ospedaliera Ospedali Riuniti Villa Sofia Cervello, Palermo, Italy; ^16^Cardio-Thoracic-Vascular Department, San Camillo-Forlanini Hospital, Rome, Italy; ^17^Cardiology Department, Garibaldi Hospital, Catania, Italy; ^18^Dipartimento Cardiotoracovascolare, Cardiology 1-Cath Lab, Coronary Care Unit (CCU), ASST Grande Ospedale Metropolitano Niguarda, Milan, Italy

**Keywords:** SARS-CoV-2, COVID-19, cancer, cardiovascular disease, cardiotoxicity, syndemic, telehealth

## Abstract

The pathophysiology of some non-communicable diseases (NCDs) such as hypertension, cardiovascular disease (CVD), diabetes, and cancer includes an alteration of the endothelial function. COVID-19 is a pulmonary and vascular disease with a negative impact on patients whose damaged endothelium is particularly vulnerable. The peculiar SARS-CoV-2-induced “endothelitis” triggers an intriguing immune-thrombosis that affects both the venous and arterial vascular beds. An increased liability for infection and an increased likelihood of a worse outcome have been observed during the pandemic in patients with active cancer and in cancer survivors. “Overlapping commonalities” between COVID-19 and Cardio-Oncology have been described that include shared phenotypes of cardiovascular toxicities such as left ventricular dysfunction, ischemic syndromes, conduction disturbances, myocarditis, pericarditis and right ventricular failure; shared pathophysiologic mechanisms such as inflammation, release of cytokines, the renin-angiotensin-aldosterone-pathway, coagulation abnormalities, microthrombosis and endothelial dysfunction. For these features and for the catalyst role of NCDs (mainly CVD and cancer), we should refer to COVID-19 as a “syndemic.” Another challenging issue is the persistence of the symptoms, the so-called “long COVID” whose pathogenesis is still uncertain: it may be due to persistent multi-organ viral attacks or to an abnormal immune response. An intensive vaccination campaign is the most successful pharmacological weapon against SARS-CoV-2, but the increasing number of variants has reduced the efficacy of the vaccines in controlling SARS-CoV-2 infections. After a year of vaccinations we have also learned more about efficacy and side-effects of COVID-19 vaccines. An important byproduct of the COVID-19 pandemic has been the rapid expansion of telemedicine platforms across different care settings; this new modality of monitoring cancer patients may be useful even in a post pandemic era. In this paper we analyze the problems that the cardio-oncologists are facing in a pandemic scenario modified by the extensive vaccination campaign and add actionable recommendations derived from the ongoing studies and from the syndemic nature of the infection.

*“COVID-19 is not a pandemic. It is a syndemic. The syndemic nature of the threat we face means that a more nuanced approach is needed if we are to protect the health of our communities” (1)*.

## Introduction

SARS-CoV-2 causes primarily pulmonary disease due to a high expression of ACE2, the entry receptor of the virus, in many epithelial cell types of the respiratory tract such as alveolar epithelial type II cells in the lungs (2, 3). ACE 2 is also expressed in extrapulmonary tissues such as nasal goblet secretory cells, cholangiocytes, colonocytes, esophageal keratinocytes, gastrointestinal epithelial cells, pancreatic β-cells, renal proximal tubule and podocytes, as documented by many studies (4–6). This widespread expression of ACE2 leads to the numerous extrapulmonary manifestations of SARS-CoV-2 infection outlined in a recent paper as thrombotic complications, myocardial dysfunction and arrhythmias, acute coronary syndromes, acute kidney injury, gastrointestinal symptoms, hepatocellular injury, hyperglycemia and ketosis, neurologic illnesses, ocular symptoms and dermatologic complications, thus making COVID-19 a truly systemic disease (7). As far as cardiovascular system is concerned, SARS-CoV-2 targets endothelial cells that abundantly express ACE2 and dysregulate the endothelium balance affecting immune competence, inflammatory balance, tight junctional barriers, hemodynamic stability and the thrombosis/fibrinolysis equilibrium (8, 9).

The COVID-19 pandemic has affected the healthcare systems throughout the world, directly by the virus-related morbidity and mortality, and by the rapid shift of resources to the infective emergency, limiting the healthcare offer for unrelated pathologies (including cardiovascular diseases and cancer). As of December 17th, 2021, patients infected by SARS-CoV-2 are over 270 millions and deaths from COVID-19 over 5 millions (10).

The first pandemic wave in the first months of year 2020 was followed by a second wave after about 6 months and, in early 2021, by a third one whose peak has been overcome in several countries thanks to the massive vaccination campaign. However, the vaccination coverage is still <50% worldwide with countries such as Russia, Venezuela and some states in the USA where 60% of the population is unvaccinated and others such as the UK and Germany with <70% of people fully vaccinated and/or not applying strict social rules such as wearing masks or limiting accesses to public events, still facing the emergency of an increasing rate of cases (11). The low vaccination coverage, the high contagiousness of new variants and the decreased efficacy of vaccines over time have contributed to the advent of the fourth wave that is now spreading all over the world at an unprecedented speed. In addition we have to struggle with new problems, such as the post-COVID syndrome (12).

The ANMCO (National Association of Italian Cardiologists) published some months ago a Position Paper (13, 14). analyzing the peculiar problems of Cardio-oncology in the COVID-19 pandemic era. In this paper we will update the previous Position Paper and recommendations according to the new scientific achievements in the field, and to the new scenario after the start of vaccination campaign.

## COVID-19, Cancer and Cardiovascular System: What We Learned in 2021

### Cancer and COVID-19

During COVID-19 pandemic, cancer patients showed a higher risk of serious events compared to non-cancer patients, including a more frequent need of invasive ventilation while admitted in the intensive care unit and higher mortality; patients treated with chemotherapy in the previous 2 weeks required more frequent admissions to the intensive care unit (15). In a recent study including more than 20,000 cancer patients a significantly increased risk of COVID-19 infection was observed among cancer patients, especially among older individuals and males; treatment with chemotherapy or immunotherapy was associated with a 2.2-fold increased risk of infection (16). Not only patients with active cancer but also cancer survivors have been shown to be more susceptible to COVID-19, in this population it has been shown that advanced age is the only risk factor for serious events (17).

In the era of immune check-point inhibitor (ICI) treatment, the question has been raised whether ICI treatment could affect protection from the virus and on the possible toxicity associated with COVID-19 vaccination. Indeed, the vaccine could “overload” the immune system and trigger a “cytokine storm,” leading to severe toxicity or even fatal events. However, in the real world the results have been controversial. A recent study of 134 cancer patients who received ICI treatment and two doses of a COVID-19 vaccine reported a similar side effect profile between cancer patients and healthy controls (18). It has been therefore hypothesized that patients receiving ICI treatment might benefit from COVID-19 vaccination and that they might also benefit from increased efficacy.

Another question has been raised regarding rituximab, an anti CD20 antibody that represents an effective treatment in many B-cell lymphomas. In patients treated with rituximab a persistent SARS-CoV-2 viraemia, an atypical COVID-19 dynamic and a persistent COVID-19 pneumonia with failure to develop anti SARS-CoV-2 antibodies have been reported, but a large meta-analysis of over 3,000 patients with hematological neoplasms did not show a correlation between concurrent treatment and worse outcome (19–22). The immunosuppressive treatment could indeed blunt the hyperinflammation and reduce the incidence of severe pneumonitis. In a more recent retrospective cohort study 12,841 immunosuppressed patients were compared to 29,386 non-immunosuppressed patients. No increased risk of mechanical ventilation or in-hospital death from the rheumatological, antineoplastic or antimetabolite therapies was observed, with the exception of patients treated with rituximab, especially female patients with cancer (23). Since rituximab-induced chronic hypogammaglobulinemia could also blunt the immune response to SARS-CoV-2 vaccination, a tailored vaccination is suggested in patients treated with rituximab (37, 38). A recent study assessed m-RNA-based COVID-19 vaccine effectiveness in patients treated with rituximab for rheumatic diseases and found that anti-CD20 treatment weakens humoral responses but does not impair T-cell responses to the vaccine (39) (Table 1).

**Table 1:** COVID-19, cancer and cardiovascular system.

**COVID-19 and Cancer**
• Potential susceptibility of the cancer population to COVID-19 and higher risk of serious events (15, 16). • Not only patients with active cancer but also cancer survivors have been shown to be more susceptible to COVID-19 (17). • Treatment with ICI is acceptable in COVID-19-infected cancer patients, except in those with severe disease (18). • Patients receiving ICI treatment might benefit from COVID-19 vaccination and they might also benefit from increased efficacy (18). • Rituximab-induced immunosuppression can lead to persistent SARS-CoV-2 viraemia and pneumonia, but a large meta-analysis did not show a worse outcome (19–22). In a more recent retrospective cohort study an increased risk of mechanical ventilation or in-hospital death was observed in patients treated with rituximab, especially female patients with cancer (23).
**COVID-19 and cardiovascular system**
• Hypertension is associated with a higher risk of severity and mortality of COVID-19 (24). • Diabetes correlates with an increased susceptibility to infection and an increased propensity for disease progression (25). • Obesity implies greater susceptibility to the virus, greater severity of disease, higher incidences of hospitalization, intensive care unit admission, and death (25). • Incidence of acute cardiac injury in COVID-19 cases is 20–40% and mortality rate is up to 10-fold higher in patients with myocardial injury at presentation (26–28). • Myocarditis is rare (<2%) (29). • Myocarditis and pericarditis after vaccination are rare events and the balance of risk and benefit is decidedly in favor of vaccination (30, 31).
**COVID-19 and Cardio-oncology**
• Overlap phenomena exist between COVID-19, tumor complications and cardiovascular effects of cancer treatments (32). • COVID-19- and anticancer drug-induced myocardial damage might have an additional effect leading to a rise in cardiovascular adverse outcomes through a “two-hit” model (33).
**Long COVID-19**
• It could be the effect of a direct result of persistent multi-organ viral attack or a chronic low grade inflammation brought about the immunomodulatory effects of the virus in the long term (34). • A persistent endotheliopathy seems to occur independently of the response to the acute phase and is accompanied by increased thrombin production (35). • It has recently been proposed that long COVID-19 may predispose to the development of cancer and accelerate its progression (36).

### COVID-19 and Cardiovascular System

Since the early studies published in China, patients hospitalized for COVID showed a high prevalence of CVD risk factors and CVD and this accounted for a more severe course of the disease and higher case fatality rates (24). The pandemic has highlighted a higher risk of severity and mortality of COVID-19 in hypertensive patients and a peculiar infectious risk in diabetic and obese patients (25). Individuals with diabetes generally suffer from chronic low-grade inflammation, which may facilitate cytokine storms, contributing to the inauspicious prognosis of COVID-19. Recently, a meta-analysis demonstrated in diabetic patients not only an increased susceptibility to the infection but also an increased disease progression of COVID-19 (40).

We are constantly learning more and more on the impact of COVID-19 on the cardiovascular system. COVID-19 has been placed in the context of the broader critical care landscape. SARS-CoV-2 infection causes myocardial injury that has a relevant role in the occurrence of severe clinical phenotypes or adverse events in affected patients. Elevated cardiac troponin is the hallmark of cardiac injury and the biomarker gives a prevalence of 20–40% of cardiac damage; myocardial injury at presentation accounts for a 10-fold increase of mortality rate (26–28).

There are many mechanisms potentially involved in the elevation of troponin in COVID-19, including thrombotic and plaque rupture events, supply-demand mismatch, direct cardiac viral toxicity, hypoxia, hypoperfusion, and tachycardia. In addition to acute myocardial infarction, troponin elevation may occur in other kinds of COVID-19 cardiovascular involvement such as viral myocarditis, cardiac damage secondary to cytokine storm, stress cardiomyopathy, heart failure (HF), pulmonary embolism, and arrhythmias (41). Myocarditis is an uncommon cause of cardiac injury, clinical and imaging markers are often suggestive of myocarditis, but the definite diagnosis requires an endomyocardial biopsy (EMB) that is rarely performed. A true autopsy- or EMB-proven diagnosis occurs in 4.5% of cases, but if we take into account some bias of autopsy studies, the percentage is even lower (42). A recent review of 22 publications with a total of 277 autopsied hearts found myocarditis in 7.2% of hearts, but a closer examination of the cases revealed that most cases were not functionally significant and the authors conclude that the true prevalence is <2% (29). Evidence of a myocarditis directly caused by the SARS-CoV-2 is scarce. Virus particles found in cardiac macrophages have been considered the result of a viremic phase or the migration of infected alveolar macrophages outside the pulmonary tissues (43–46). The risk of mortality and adverse events follows a continuous linear trend with the degree of troponin increase; therefore, troponin measurement has been incorporated into routine clinical practice in hospitalized COVID- 19 patients. A recent study has challenged previously acquired certainties, myocardial damage in severe COVID-19 has been shown to be driven by underlying comorbidities, advanced age, and multisystem organ dysfunction. These findings raise a new question: does myocardial damage evidenced by troponin represent a mediator or a marker of adverse outcome? (47).

Furthermore, in an international, retrospective multicenter study of echocardiographic findings in more than 300 patients admitted with COVID-19, a significantly higher risk of in-hospital mortality was observed only in patients with troponin elevation and echocardiographic abnormalities, not just elevated troponin (48).

During the early phase of the pandemic, there was initially theoretical uncertainty about the safety of using angiotensin-converting enzyme inhibitors (ACEIs)/angiotensin II receptor blockers (ARBs) in patients with COVID-19. ACE2 is a receptor for SARS-CoV-2, therefore concern was initially raised in the medical and scientific community that the use of ACEIs and ARBs could result in increased mortality and severity of COVID-19. Since 12-day administration of losartan or both losartan and lisinopril induced an increase in cardiac Angiotensin Converting Enzyme 2 (ACE2) mRNA and in cardiac membrane ACE2 activity in rats (49), it was hypothesized that ACEIs and ARBs could increase the entrance receptors for SARS-CoV-2 infection leading to a more severe infection and higher mortality. Subsequent studies have allayed initial fears, demonstrating not only the potential benefit of ACEI/ARB treatment in hospitalized patients with hypertension and COVID-19, but also a reduction in COVID-19 all-cause mortality in treated vs. untreated patients (50). A special report described the uncertain effect of renin-angiotensin-aldosterone system (RAAS) inhibition in humans due to the paucity of studies regarding the effect of RAAS inhibition on ACE2 expression confirming that RAAS inhibitors should be continued in hypertensive patients at risk for or with COVID-19 (51). A recent meta-analysis of 26 studies confirmed that treatment with ACEIs and ARBs compared with other antihypertensive drugs or no treatment was associated with reduced mortality as well as a lower risk of ventilatory support among COVID-19-infected hypertensive patients (52).

Major scientific Societies have provided recommendations in favor of continued treatment with ACEIs and ARBs in patients with hypertension, HF, and ischemic heart disease (53–55) (Table 1).

### Myocarditis and Pericarditis After Vaccination for COVID-19

Although the physiopathology of myocarditis is still unclear, it has been hypothesized that vaccine mRNA can be identified as an antigen by the immune system that activates pro-inflammatory cascades and immunological pathways that may have a relevant role in the development of a systemic reaction of which myocarditis is an important component. Another mechanism could be related to molecular mimicry between the coronavirus spike protein and self-antigens whereby a cross-reaction may occur between antibodies against SARS-CoV-2 spike glycoproteins and structurally similar peptide protein sequences, such as α-myosin (56). A possible association between COVID-19 mRNA vaccines and myocarditis, mainly in younger male individuals within a few days after the second vaccination, has been recently reported by the Centers for Disease Control and Prevention, with an incidence of ~4.8 cases per 1 million (30). According to a recent report on 2,000,287 vaccinated subjects, myocarditis developed in 20 young patients, a median of 3.5 days after vaccination, especially after the second dose of vaccine. Pericarditis affected 37 patients with a median onset of 20 days after the most recent vaccination (31). Despite these rare events, the balance of risk and benefit is decidedly in favor of vaccination against COVID-19 (Table 1).

### Cardio-Oncology and COVID-19

In the cardio-oncology population, additional diagnostic complexity has been observed due to “overlap” phenomena between COVID-19, tumor complications, and cardiovascular side effects of cancer treatments. Cardiovascular toxicities shared by COVID-19 and cardio-oncology include myocardial injury, cardiomyopathy, myocarditis, pericarditis, ischaemia, conduction disturbances involving immune system activation, cytokine release syndrome, arterial and venous coagulopathy (32). It should be emphasized that in this population, the increased troponin assumes an even more intriguing significance since it may be also indicative of subclinical cardiotoxicity induced by treatments with anthracyclines and/or anti-HER2 agents, and it can be observed in patients receiving tyrosine kinase inhibitors at high prothrombotic risk or fluoropyrimidines. Studies are needed to define whether cardiac injury deriving from SARS-CoV-2 infection and from anticancer drugs might have an additional effect leading to a rise in cardiovascular adverse outcome through a “two-hit” model, both in cancer patients and survivors (33). A recent analysis of an AHA COVID-19-based CVD registry did not show a significant difference of in-hospital mortality among cancer patients with or without preexisting CVD, on the other hand (and in contrast to previous studies), a strong independent association of oncologic treatment with in-hospital morbidity was observed. The combination of these data provides the cue for a delicate reflection that should involve both oncologists and cardiologists inviting them to share with their patients the definition of the optimal timing of anti-cancer therapies according to the necessity to cope with limited health resources and an infection breakdown, obviously balancing the possible need for urgent therapy according to cancer type and cancer status (57) (Table 1).

### Post-acute COVID-19 Syndrome “(Long COVID)”

Several outpatients' clinics are flooded by patients affected by long-lasting symptoms: the so-called “long COVID” syndrome. This syndrome is better defined as “post-acute COVID-19 syndrome (PACS)” if the symptoms last more than 3 weeks and “chronic COVID-19” if they last more than 12 weeks (58, 59). The National Institutes of Health has defined “long COVID” as post-acute sequelae of SARS-CoV-2 infection (PASC) (60). Initial reports, currently confirmed, have highlighted the following residual effects of SARS-CoV-2 infection: fatigue, dyspnea, chest pain, cognitive impairment, arthralgia, and decline in quality of life (61). Symptomatic tachycardia, either presenting as postural orthostatic tachycardia syndrome or inappropriate sinus tachycardia, is also frequently reported in post-acute COVID-19 syndrome (62). All these symptoms may pose problems of differential diagnosis with symptoms originating from a primary cardiovascular problem. The overdrive of host immunity in response to the virus may contribute to severe disease. Long COVID-19 could be a chronic low-grade inflammation brought about by the immunomodulatory effects of the virus in the long-term (34). It has recently been proposed that long COVID-19 may predispose to the development of cancer and accelerate its progression. The hypothesis comes from an increased evidence of a relevant role of SARS-CoV-2 in modulating oncogenic pathways, promoting chronic low-grade inflammation and causing tissue damage. Responses in COVID-19 patients are governed by proinflammatory cytokines (IL-1, IL-6, IL-8, and TNF-α), which are also drivers of oncogenesis.

Hypoxia due to inflammation can induce oxidative stress that synergistically with chronic inflammation can lead to DNA damage and subsequent tumorigenesis (36). A recent study has shown a frequent prolonged activation of endothelial cells (up to 10 weeks after acute SARS-CoV-2 infection) and this sustained endotheliopathy seems to be independent from the response to the acute phase and is accompanied by increased thrombin production (35). These data open a new scenario that raises a question about the stratification of thrombotic risk after the resolution of the acute infection and the possible need for prolonged thromboprophylaxis. Multidisciplinary collaboration is essential to provide appropriate outpatient care for COVID-19 survivors (Table 1).

### Cardio-Oncological Counseling in COVID-19 Pandemic

#### The Very Early Phase

Shortly after the pandemic spread we learned that patients with cardiovascular disease and cancer were at a higher risk of acquiring the infection and of experiencing poorer outcomes (63). Cardio-oncology focuses on the intersection of two pathologies that both affect, by definition, “fragile” patients. For these reasons Cardio Oncology Services have faced a series of issues, which have influenced both the clinical and organizational areas:

- The subgroups most at risk seem to be patients on active therapy, in particular those with signs/symptoms attributable to cardiotoxicity; patients being treated with immunosuppressive drugs and patients who have undergone autologous or allogeneic haematopoietic stem-cell transplantation (64–66). For this reason, the absolute need to protect these subgroups of patients from the possibility of contracting COVID-19 has emerged since the very beginning.
- Cancer patients with or without pre-existing cardiovascular disease were in any case indirectly involved in the profound reorganization of both territorial and hospital health services that the pandemic urged to make, as well as by the reallocation of human and structural resources to the management of COVID-19 patients. This has led to the postponement and reprogramming of diagnostic tests and treatments with a clear impact on cancer outcome (67, 68).

#### What Have We Learned so Far?

The COVID-19 pandemic has represented and still represents a unique opportunity for a reasoned review on the appropriateness of our clinical cardio-oncology practice which still lacks shared guidelines and is frequently anchored to local habits (69). During pandemic our watchwords have become appropriateness and optimization of therapeutic and follow-up paths. We therefore learned that risk stratification of our cardio-oncology patients played a key role. Identifying truly low-risk patients makes it possible to concentrate the limited resources available on patients at higher cardiological risk, for whom the deferral of clinical and instrumental controls could actually have negative consequences.

Recommendations for a modified screening and monitoring schedule to detect cardiac dysfunction, and judicious use of multimodality imaging and biomarkers to identify heart involvement during pandemic are actually available from three international groups (70–72) and have been variously applied in order to minimize the outpatient accesses to hospital. The central issue is to obtain baseline LVEF assessment and to keep standard monitoring by means of trans-thoracic echocardiography only in those patients considered to be at high risk for cardiotoxicity and to reserve additional imaging to selected cases.

The COVID-19 pandemic has propelled the use of telemedicine because it can be accessed by people directly from home and may reduce the probability of viral transmission by limiting hospital accesses and interpersonal contacts. Over the course of <1 year, many centers have shifted the majority of follow-up cancer care to virtual modality, a dramatic transformation in the way our patients' care is delivered. The video-visit volume at the University of California, San Francisco Comprehensive Cancer Center expanded from <20 to 72% in a brief time at the beginning of the pandemic (73). In the first months of the pandemic a national survey evaluated the impact of COVID-19 on Canadian medical oncologists, 82% of medical oncologists reported the implementation of telemedicine for many cancer patients: telephone call was utilized in 100% of cases, videoconferencing was used in 42% and e-mail in 12% of cases (74). An early implementation of Virtual Care was reported as feasible in a high volume cancer center in Ontario Canada from March to May 2020 with a preserved quality of care (75). Even though multiple barriers, including cost-effectiveness, security of communication links for personal data (including health), limitations/unreliability of internet connections, concerns regarding the impact of telemedicine on doctor-patient relationship, liability and legal issues, time constraints, and financial (e.g., billing) obstacles have slowed progress of telemedicine, the data collected in this period make telemedicine a valuable component of our clinical practice that will last beyond the pandemic (76, 77) (Table 2).

**Table 2:** The four pillars of counseling.

• Limitation of hospital accesses • Spread of telemedicine • Restriction of imaging sessions • More extensive and reasoned use of biomarkers

### Cardio-Oncological Consulting in Outpatients

For cardio-oncological patients, a first distinction must be made between the outpatient and the hospital level, with a further differentiation, between COVID-free “Cancer Centers” and general hospital. At first, the only effective strategy to contain the spread of the disease appeared to be social distancing (78) and, for cancer patients, this translates into the need to limit hospital access to selected cases.

In cancer patients with no previous CVD, an accurate risk stratification could be based on the anamnestic criteria only, by a shared cardiological and oncological evaluation. The cardiologist's task is to provide the oncologist with simple flowcharts to identify low-risk patients, for whom cardiological consultation in presence is not necessary, once a baseline electrocardiogram and a pretreatment echocardiogram (if needed) have been acquired. For patients with known CVD it is not always possible to safely defer or to skip cardiological checks.

In order to restrict accesses to hospital to high-risk patients only, an appropriate triage for patients with new cancer diagnosis and cancer survivors is mandatory and telemedicine can fulfill this purpose. A first approach can include a cardiologist's telephone contact aimed at ascertaining the clinical stability of the patient. This evaluation can possibly be integrated by telemedicine tools, as the transmission of the instrumental tests held by the patient. This preliminary “virtual visit” assesses cardiac risk; if the risk is high an “in person” cardio-oncology visit is suggested, if the cardiac risk is low a “virtual” cardio-oncology visit is planned (72). Telemedicine is indeed in the spotlight, especially in the USA, where in 2020 Congress approved a regulation (79) which allows certain providers to charge Medicare for some services provided through telemedicine. In spring 2020, there was an increasing use of online platforms, as a tool to keep patients out of the hospital (80, 81). However, in many countries the regulatory framework and the possibility of reimbursement for telemedicine activities are still very poor. Furthermore, the unavailability of technology and the lack of digital literacy could accentuate the inequalities in access to specialized medical care. And this is an issue that affects mainly the most disadvantaged population groups, such as patients of low socioeconomic status, the elderly and immigrants (82). Actually “*equitable”* care is one of the 6 quality dimensions of telehealth interventions provided by the Institute of Medicine's report: “*care that is safe, effective, patient-centered, timely, efficient and equitable”* (83).

As far as telemedicine in the cardio-oncology field is specifically concerned, an international survey conducted between March and April 2020, which involved over 1,400 cardiologists and oncologists from 43 countries, showed a rapid growth in telemedicine already in the first months of the pandemic. Of note, cardiologists more often than oncologists reported the need to cancel or postpone elective visits or treatments, and that can partly be explained by the fact that cardiologists were more often directly involved in the care of COVID-19 patients (84) (Table 2).

### Cardio-Oncological Counseling in Hospitalized Patients

In this context too, the primary need is to protect “fragile” patients, minimizing the chances of contagion. Within non-COVID-free general hospitals, it is necessary to provide and organize protected pathways for cancer patients. More extensive use of biomarkers to reduce imaging sessions and the use of portable hardware (POCUS, point-of care ultrasound) could find application in hospitalized patients even more than in outpatients. In hospitalized patients, a problem that could arise from a wider use of biomarkers is represented by the differential diagnosis between manifestations of cardiotoxicity and a possible SARS-CoV-2-related cardiac involvement in the course of infection, considering, however, that the former is much more frequent than the latter. Finally, the clinical and instrumental pre-surgery operative cardiological evaluation of patients to be sent to oncological surgery which, especially in Cancer Centers, is widely used, should even more be limited to cases in which the results of the consultation is able to modify the surgical choices and/or treatment (85) (Table 2).

## Adapted Cardiac Monitoring in the Vaccination Era

Basal cardiovascular screening and on-treatment monitoring in cancer patients receiving potentially cardiotoxic therapies are of fundamental importance to reduce cardiac toxicity and improve outcome (87). The costs of pandemic both in terms of the direct impact on healthcare system and by the huge amount of cumulated backlogs in elective diagnostic procedures impose a deep reflection about how to improve both sustainability and equity in healthcare (88). The need to recover unperformed cardiac evaluations/tests together with an increasing number of tests required by new diagnoses suggests a common strategy to harmonize cardiac surveillance protocols avoiding unnecessary tests and reducing the frequency of examinations under certain circumstances. The modifications applied to cardiac monitoring protocols during the first wave of pandemic could offer some solutions to be implemented even in the vaccination era. The central idea of a careful stratification of the risk of cardiac toxicity should get more and more relevance. Limited healthcare resources should be focused on people with a higher baseline risk of toxicity and in this setting the frequency of cardiac consultations should be kept unchanged. On the other hand we could safely increase the time period between visits in very-low and low risk population. An additional solution could be the relocation of some routine activity in low-risk patients in out-of-hospital (OOH) facilities in close collaboration with general practitioners. Baseline and on-treatment cardiac monitoring are ideal candidates to test this new risk-based model.

### General Considerations

The proposed post-COVID recommendations on cardiac monitoring are focused on the general surveillance schedule for patients receiving anthracyclines and anti-HER2 agents. Cardiac surveillance in those cancer patients with a higher probability to develop cardiotoxicity and/or when an appropriate early cardiological treatment is advisable to avoid delays or interruptions of anticancer treatment program must continue unchanged. Cardiological visits should coincide with cancer therapy administration to reduce the need of hospital accesses. Cardiac imaging monitoring should be focused on the predicted toxicity. Alternative imaging techniques [as computed tomography (CT) scan, cardiac magnetic resonance, and nuclear medicine techniques] (89, 90), should be reserved to selected cases based on cardio-oncologist consultation only.

In subsequent visits in asymptomatic low-risk patients, it could be reasonable to reduce the general duration of echo examination. In centers with specific expertise in monitoring cardiac toxicity by means of serial troponin and/or brain natriuretic peptide, the frequency of imaging could be reduced in asymptomatic patients with persistent normal values (<99th percentile) of biomarkers given their high negative predictive value (91). In those centers where biomarkers are routinely tested, we suggest to use routine cancer treatment-related blood draws to minimize exposures. Table 3 summarizes recommendations for an adapted risk-based imaging and clinical assessment schedule.

**Table 3:** Proposal for a risk-based approach to planned cardiac monitoring during anthracycline and trastuzumab treatment in the vaccination era.

**Treatment**	**Recommendations before pandemic**	**Recommendation during pandemic**	**Recommendation during vaccination**
Anthracyclines: basal evaluation	• Cardiological visit only in intermediate and high-risk patients^*^ • Echocardiography to all patients	• Cardiological visit only in high-risk patients^*^ • Echocardiography only in high-risk patients^*^	• Cardiological visit only in high-risk patients^*^ • Echocardiography only in high-risk patients^*^
Anthracyclines: on treatment	• Echocardiography at mid-cycle if high CV risk • Echocardiography at the end of treatment to all patients	• No screening in asymptomatic patients • Echocardiography if high-dose RT, high cumulative anthracycline dose (>400 mg/m^2^) or with doses of 250 mg/m^2^ in presence of CV risk factors or cardiopathy	• Echocardiography at the end of treatment to all patients (OOH) • Early assessment if high-dose RT, high cumulative anthracycline dose (>400 mg/m^2^) or with doses of 250 mg/m^2^ in presence of CV risk factors or cardiopathy
Anthracyclines: follow-up	• If no cardiotoxicity echocardiography at 6– 12 months and after 2–3–5 years • If cardiotoxicity echocardiography at 3–6–12 months and each year until 5 years	• In asymptomatic patients defer the echo-imaging	• If no cardiotoxicity echocardiography at 12 months and after 2–5 years in intermediate and high-risk patients^*^ • If no cardiotoxicity echocardiography at 12 months in low-risk patients^**^ (OOH) • If cardiotoxicity echocardiography at 3–6–12 months and each year until 5 years
Trastuzumab: basal evaluation	• Echocardiography to all patients	• Echocardiography only in high-risk patients	• Echocardiography only in intermediate and high-risk patients
Trastuzumab: during treatment	• If LVEF is normal, echocardiography every 3 months. • If LVEF 40–49%, optimize HF therapy. Continue treatment if LVEF stable after 4 weeks and repeat echocardiography after 4 weeks. • If LVEF <40% stop treatment, optimize HF therapy and evaluate after 4 weeks	• In low-risk^**^ patients with no previous anthracyclines, echocardiography at 6–12 months; if metastatic disease echocardiography every 6 months • In high-risk patients^*^ echocardiography every 3 months • If LV dysfunction or signs and symptoms of HF follow pre-pandemic recommendations	• In low-risk^**^ patients with no previous anthracyclines, echocardiography every 6 months (OOH) • In high-risk patients^*^ echocardiography every 3 months • If LV dysfunction during treatment or signs and symptoms of HF follow pre-pandemic recommendations
Trastuzumab: follow-up	• The same as anthracyclines	• If asymptomatic defer the echo imaging	• If no cardiotoxicity echocardiography at 12 months and after 2 years in intermediate and high-risk patients^*^ • If no cardiotoxicity echocardiography at 12 months in low-risk patients^**^ (OOH) • If cardiotoxicity echocardiography at 3–6–12 months and each year until 5 years

*Adapted from Calvillo-Arguelle et al. (86) and Bisceglia et al. (13, 14) for before pandemic and during pandemic sections. CV, cardiovascular; RT, radiotherapy; OOH, out-of-hospital; LVEF, left ventricular ejection fraction*.

* *Two or more of the following risk factors: age ≥60 years, cardiopathy, high-dose radiotherapy, ≥2 cardiovascular risk factors, high-dose anthracyclines*.

** *No risk factors*.

### Baseline Evaluation of Cancer Patient

#### Anthracyclines

Baseline cardiac imaging should be offered to patients with a history of significant CVD, with signs or symptoms of cardiac dysfunction, with two or more cardiovascular (CV) risk factors for cardiotoxicity (age ≥60 years, hypertension, diabetes mellitus, dyslipidaemia, smoking, or obesity). If the execution of baseline evaluation is not feasible before treatment, it may be reasonable to postpone it during treatment in asymptomatic and low-risk patients. For adult patients whose only risk factor is a planned high cumulative doxorubicin dose (≥250 mg/m^2^), it may be reasonable to delay imaging until this threshold dose is reached or at the end of treatment (86).

#### Trastuzumab

Basal screening should be reserved to patients with a known CVD, with signs or symptoms of cardiac dysfunction, with 2 or more CV risk factors for cardiotoxicity (age ≥60 years, hypertension, diabetes mellitus, dyslipidaemia, smoking, or obesity), prior exposure to anthracyclines. In patients without valvular disease and a normal ventricular function (LVEF ≥55%) assessed in the previous 6 months, it is reasonable to avoid basal evaluation (86).

### Surveillance During Treatment

#### Anthracyclines

The majority of cardiac dysfunction observed during anthracyclines therapy are mild and moderate with a very low mortality rate. Therefore, in the general population it could be reasonable to delay routine imaging during anthracycline therapy and perform a single final evaluation except for the following cases: signs and symptoms of HF or anthracycline dosages >400 mg/m^2^ or cardiac risk factors and need for anthracycline therapy >250 mg/m^2^, especially when there is a potential clinical impact of cardio-protective strategies. In those centers that routinely use biomarkers, cardiological evaluation should be performed in case of significant rise of biomarkers (86).

#### Trastuzumab

In the adjuvant setting, asymptomatic women without CV risk factors and not previously treated with anthracycline may undergo echocardiography at a reduced schedule of evaluation at 6 and 12 months only. In the metastatic setting, an echocardiogram could be performed every 6 months in the first year; beyond first year cardiac imaging may be deferred in asymptomatic patients. In patients with risk factors for cardiotoxicity (prior anthracycline exposure, CV risk factors) it is necessary to keep cardiac surveillance every 3 months. Patients with borderline ejection fraction (EF) 50–55% or reduced LVEF or with signs or symptoms of HF must continue to have a closer imaging schedule. In those centers that routinely use biomarkers, cardiological evaluation should be performed in case of significant rise of biomarkers (86).

#### Follow-Up

Routine cardiac follow-up in asymptomatic survivors of pediatric, adolescent, and young adult cancers could be moved to OOH facilities. Immediate cardio-oncological consultation will be provided in case of symptoms or signs of toxicity.

### Perspectives

COVID-19 pandemic has forced the cardio-oncology community to make a re-evaluation on how to deliver the best clinical care. In addition to the aforementioned leading role of the appropriateness issue, one of the most important byproducts of COVID-19 pandemic has been the growth of telemedicine platforms across different care settings. In an era of digital technologies in many aspects of our life, COVID-19 has accelerated digital transformation, this impressive transition has been called “techcelleration” (92). For clinicians this paradigm shift from an interactive empathic “face to face” visit to a mere decoding of data from a smart screen has been challenging, some of them accept these changes, but others are troubled by this profound transformation.

Moreover, multi-organ point-of-care ultrasound (PoCUS), including lung ultrasound (LUS) and focused cardiac ultrasound (FoCUS), has impacted greatly on the management of COVID-19 patients both at triage and at subsequent clinical management. An expert panel has developed a consensus document on the use of PoCUS in COVID-19 patients. PoCUS was useful in nine clinical domains (diagnosis of SARS-CoV-2 infection, initial triage and risk stratification, diagnosis of Covid-19 pneumonia, diagnosis of cardiovascular disease, screening for venous thromboembolic disease, respiratory support strategies, management of fluid therapy, clinical monitoring of patients with COVID-19, and infection control to reduce the environmental spread of infection and risk of infection for health care providers) (93).

In the future we will also have to be able to minimize the disparities in accesses to care that the pandemic has highlighted. This will enable us to better face future pandemics and limit their spread using models that have proven effective against COVID-19, without losing contact with our patients and compromising the effectiveness of cancer and cardiological treatments. The rapidly accumulating data and patients' follow-up we are accompanying through the storm of the COVID-19 pandemic will allow us to refine our approach to what increasingly resembles “precision cardio-oncology.” The “digital future is now” is the warning of the editors of JACC Heart Failure (92), therefore we must be ready to support the valuable components of this transition and their “*potential for a better tomorrow”* (92).

Finally, the tremendous impact of the virus on CVD and cancer patients should fuel a vigorous campaign to implement healthy lifestyles that will reduce the burden of CVD and cancer, improve the health of our planet and eventually stop the syndemic.

## Author Contributions

IB, MC, GG, FT, and CL wrote sections of the manuscript. All authors contributed to the conception of the work, manuscript revision, read, and approved the submitted version.

## Conflict of Interest

The authors declare that the research was conducted in the absence of any commercial or financial relationships that could be construed as a potential conflict of interest.

## Publisher's Note

All claims expressed in this article are solely those of the authors and do not necessarily represent those of their affiliated organizations, or those of the publisher, the editors and the reviewers. Any product that may be evaluated in this article, or claim that may be made by its manufacturer, is not guaranteed or endorsed by the publisher.
