# Corrigendum: Cardio-Oncology in the COVID Era (Co & Co): The Never Ending Story

**DOI:** 10.3389/fcvm.2022.903766

**Published:** 2022-05-13

**Authors:** Irma Bisceglia, Maria Laura Canale, Giuseppina Gallucci, Fabio Maria Turazza, Chiara Lestuzzi, Iris Parrini, Giulia Russo, Nicola Maurea, Vincenzo Quagliariello, Stefano Oliva, Stefania Angela Di Fusco, Fabiana Lucà, Luigi Tarantini, Paolo Trambaiolo, Antonella Moreo, Giovanna Geraci, Domenico Gabrielli, Michele Massimo Gulizia, Fabrizio Oliva, Furio Colivicchi

**Affiliations:** ^1^Integrated Cardiology Services, Cardio-Thoracic-Vascular Department, Azienda Ospedaliera San Camillo Forlanini, Rome, Italy; ^2^Cardiology Department, Nuovo Ospedale Versilia Lido di Camaiore Lucca, Camaiore, Italy; ^3^Cardio-Oncology Unit, Istituto di Ricovero e Cura a Carattere Scientifico (IRCCS), Referral Cancer Center of Basilicata, Rionero in Vulture, Italy; ^4^Cardiology Department, National Cancer Institute Foundation, Milan, Italy; ^5^Azienda Sanitaria Friuli Occidentale (ASFO) Cardiac and Cardio-Oncologic Rehabilitation Service at Centro di Riferimento Oncologico (CRO), National Cancer Institute, Aviano, Italy; ^6^Cardiology Department, Ospedale Mauriziano Umberto I, Turin, Italy; ^7^Cardiovascular and Sports Medicine Department, Azienda Sanitaria Universitaria Giuliano Isontina (ASUGI) Trieste, Trieste, Italy; ^8^Cardiology Department, G. Pascale National Cancer Institute Foundation (IRCCS), Naples, Italy; ^9^Cardiology Department, G. Pascale National Cancer Institute Foundation (IRCCS), Naples, Italy; ^10^Cardio-Oncology Department, Istituto Tumori Giovanni Paolo II, Bari, Italy; ^11^Clinical and Rehabilitation Cardiology Department, Ospedale San Filippo Neri, Rome, Italy; ^12^Cardiology Department, Grande Ospedale Metropolitano Bianchi Melacrino Morelli, Reggio Calabria, Italy; ^13^Divisione di Cardiologia, Arcispedale S. Maria Nuova, Azienda Unità Sanitaria Locale-IRCCS di Reggio-Emilia, Reggio Emilia, Italy; ^14^Cardiology Department, Sandro Pertini Hospital, Rome, Italy; ^15^A. De Gasperis Cardio Center, Aziende Socio Sanitarie Territoriali (ASST) Grande Ospedale Metropolitano Niguarda, Milan, Italy; ^16^Cardiology Unit, Azienda Ospedaliera Ospedali Riuniti Villa Sofia Cervello, Palermo, Italy; ^17^Cardio-Thoracic-Vascular Department, San Camillo-Forlanini Hospital, Rome, Italy; ^18^Cardiology Department, Garibaldi Hospital, Catania, Italy; ^19^Dipartimento Cardiotoracovascolare, Cardiology 1-Cath Lab, Coronary Care Unit (CCU), ASST Grande Ospedale Metropolitano Niguarda, Milan, Italy

**Keywords:** SARS-CoV-2, COVID-19, cancer, cardiovascular disease, cardiotoxicity, syndemic, telehealth

In the published article, there was an error in affiliation 13. Instead of “Cardiology Department, Santa Maria Nuova Hospital, Reggio Emilia, Italy,” it should be “Divisione di Cardiologia, Arcispedale S. Maria Nuova, Azienda Unità Sanitaria Locale-IRCCS di Reggio-Emilia, Reggio Emilia, Italy.”

The authors apologize for this error and state that this does not change the scientific conclusions of the article in any way. The original article has been updated.

## Publisher's Note

All claims expressed in this article are solely those of the authors and do not necessarily represent those of their affiliated organizations, or those of the publisher, the editors and the reviewers. Any product that may be evaluated in this article, or claim that may be made by its manufacturer, is not guaranteed or endorsed by the publisher.

