# Corrigendum: Cardio-oncology in the COVID Era (Co & Co): The never-ending story

**DOI:** 10.3389/fcvm.2023.1169176

**Published:** 2023-03-09

**Authors:** Irma Bisceglia, Maria Laura Canale, Giuseppina Gallucci, Fabio Maria Turazza, Chiara Lestuzzi, Iris Parrini, Giulia Russo, Nicola Maurea, Vincenzo Quagliariello, Stefano Oliva, Stefania Angela Di Fusco, Fabiana Lucà, Luigi Tarantini, Paolo Trambaiolo, Antonella Moreo, Giovanna Geraci, Domenico Gabrielli, Michele Massimo Gulizia, Fabrizio Oliva, Furio Colivicchi

**Affiliations:** ^1^ Integrated Cardiology Services, Cardio-Thoracic-Vascular Department, Azienda Ospedaliera San Camillo Forlanini, Rome, Italy; ^2^ Cardiology Department, Nuovo Ospedale Versilia Lido di Camaiore Lucca, Camaiore, Italy; ^3^ Cardio-Oncology Unit, Istituto di Ricovero e Cura a Carattere Scientifico (IRCCS), Referral Cancer Center of Basilicata, Rionero in Vulture, Italy; ^4^ Cardiology Department, Fondazione IRCCS Istituto Nazionale dei Tumori, Milan, Italy; ^5^ Azienda Sanitaria Friuli Occidentale (ASFO) Cardiac and Cardio-Oncologic Rehabilitation Service at Centro di Riferimento Oncologico (CRO), National Cancer Institute, Aviano, Italy; ^6^ Cardiology Department, Ospedale Mauriziano Umberto I, Turin, Italy; ^7^ Cardiovascular and Sports Medicine Department, Azienda Sanitaria Universitaria Giuliano Isontina (ASUGI) Trieste, Trieste, Italy; ^8^ Cardiology Department, G. Pascale National Cancer Institute Foundation (IRCCS), Naples, Italy; ^9^ Cardio-Oncology Department, Istituto Tumori Giovanni Paolo II, Bari, Italy; ^10^ Clinical and Rehabilitation Cardiology Department, Ospedale San Filippo Neri, Rome, Italy; ^11^ Cardiology Department, Grande Ospedale Metropolitano Bianchi Melacrino Morelli, Reggio Calabria, Italy; ^12^ Divisione di Cardiologia, Arcispedale S. Maria Nuova, Azienda Unità Sanitaria Locale- IRCCS di Reggio-Emilia, Reggio Emilia, Italy; ^13^ Cardiology Department, Sandro Pertini Hospital, Rome, Italy; ^14^ A. De Gasperis Cardio Center, Aziende Socio Sanitarie Territoriali (ASST) Grande Ospedale Metropolitano Niguarda, Milan, Italy; ^15^ Cardiology Unit, Azienda Ospedaliera Ospedali Riuniti Villa Sofia Cervello, Palermo, Italy; ^16^ Cardio-Thoracic-Vascular Department, San Camillo-Forlanini Hospital, Rome, Italy; ^17^ Cardiology Department, Garibaldi Hospital, Catania, Italy; ^18^ Dipartimento Cardiotoracovascolare, Cardiology 1-Cath Lab, Coronary Care Unit (CCU), ASST Grande Ospedale Metropolitano Niguarda, Milan, Italy

**Keywords:** SARS-CoV-2, COVID-19, cancer, cardiovascular disease, cardiotoxicity, syndemic, telehealth

A Corrigendum on Cardio-oncology in the COVID Era (Co & Co): The never ending story By Bisceglia I, Canale ML, Gallucci G, Turazza FM, Lestuzzi C, Parrini I, Russo G, Maurea N, Quagliariello V, Oliva S, Di Fusco SA, Lucà F, Tarantini L, Trambaiolo P, Moreo A, Geraci G, Gabrielli D, Gulizia MM, Oliva F and Colivicchi F (2022) Front. Cardiovasc. Med. 9:821193. doi: 10.3389/fcvm.2022.821193

**Incorrect Affiliation**

In the published article, there was an error in affiliations [4] and [9]. Affiliation 4 should be “4. Cardiology Department, Fondazione IRCCS Istituto Nazionale dei Tumori, Milan, Italy”. Instead of “4. Cardiology Department, National Cancer Institute Foundation, Milan, Italy”.

Affiliation 9 should be “9. Cardio-Oncology Department, Istituto Tumori Giovanni Paolo II, Bari, Italy”, Instead of “Cardiology Department, G. Pascale National Cancer Institute Foundation (IRCCS), Naples, Italy”.

The authors apologize for this error and state that this does not change the scientific conclusions of the article in any way. The original article has been updated.

